# Mortality following proximal femoral fractures in elderly patients: a large retrospective cohort study of incidence and risk factors

**DOI:** 10.1186/s12891-023-06825-9

**Published:** 2023-08-30

**Authors:** Soroush Baghdadi, Maryam kiyani, Seyyed Hadi kalantar, Samira Shiri, Omid Sohrabi, Shahabaldin Beheshti Fard, Sina Afzal, Seyyed Saeed Khabiri

**Affiliations:** 1 Division of Orthopaedics, Children’s Hospital of Philadelphia, Philadelphia, PA USA; 2 School of Medicine, Kermanshah University of Medical Sciences, Kermanshah, Iran; 3 Joint Reconstruction Research Center, Tehran University of Medical Sciences, P.O. box: 1419733141, Tehran, Iran; 4 Clinical Research Development Centre, Taleghani and Imam Ali Hospital, Kermanshah University of Medical Sciences, Kermanshah, Iran; 5 Department of Orthopedic Surgery, School of Medicine, Shahid Beheshti University of Medical Sciences, Tehran, Iran

**Keywords:** Proximal femoral fractures, Mortality, Survival analysis, Outcome Assessment, Iran

## Abstract

**Background:**

Global prevalence of osteoporosis and fragility fractures is increasing due to the aging population. Proximal femoral fractures are among the most common orthopedic conditions in elderly that significantly cause health deterioration and mortality. Here, we aimed to evaluate the mortality rates and risk factors, besides the functional outcomes after these injuries.

**Methods:**

In a retrospective cohort study, all patients admitted with a femoral neck or intertrochanteric fracture between 2016 and the end of 2018 were enrolled in this study. Medical records were reviewed to include patients over 60 years of age who had a proximal femoral fracture and had a complete medical record and radiographs. Exclusion criteria included patients with pathological fractures, cancer under active treatment, follow-up loss, and patient access loss. Demographic and clinical features of patients alongside the details of fracture and patient management were recorded and analyzed. In-hospital and post-discharge mortalities due to included types of fractures at one and 12 months were the primary outcome. Modified Harris Hip Scores (mHHS) was the measure of functional outcome.

**Results:**

A total of 788 patients including 412 females (52.3%) and 376 males (47.7%) with a mean age of 76.05 ± 10.01 years were included in this study. Among patients, 573 (72.7%) had an intertrochanteric fracture, while 215 (27.3%) had a femoral neck fracture, and 97.1% of all received surgical treatment. With a mean follow-up of 33.31 months, overall mortality rate was 33.1%, and 5.7% one-month and 20.2% 12-months rates. Analysis of 1-month mortality showed a significant mortality difference in patients operated after 48 h of fracture (p = 0.01) and in patients with American Society of Anesthesiologists (ASA) scores of 3–4 compared to ASA scores of 1–2 (p = 0.001). One-year mortality data showed that the mortality rate in femoral neck fractures was lower compared to other types of fracture. Surgical delay of > 48 h, ASA scores of 3–4, and treatment by proximal femoral plate were associated with shorter survival. The overall mean mHHS score was 53.80 ± 20.78.

**Conclusion:**

We found several risk factors of mortality, including age ≥ 80 years, a > 48-hour delay to surgery, and pre-operative ASA scores of 3–4 in patients with proximal femoral fracture. Furthermore, the use of a proximal femoral plate was a significant risk factor for mortality and lower mHHS scores.

## Background

Due to the aging population, the worldwide prevalence of osteoporosis is rising, and correspondingly, the incidene of fragility fractures are increasing [1]. National Osteoporosis Foundation estimates that over 53 million individuals have or are at a high risk of osteoporosis in the United States, and by 2040, the total yearly expenses of caring for fragility fractures will increase to over $95 billion [2]. While spine and wrist fractures are also common, proximal femoral fractures are the most common fragility fractures, accounting for up to 40% of all osteoporotic fractures [3]. Thus, proximal femoral fractures represent a substantial proportion of fracture-related admissions and impose a significant burden to healthcare systems worldwide, accounting for up to 72% of all fracture-related costs in the United States [4, 5]. Similar data have been reported from Europe, in which the cost of treating fragility fracture was €37.5 billion in 2017 [6].

Proximal femoral fractures significantly contribute to health deterioration and long-term morbidity and mortality. The arduous rehabilitation, functional decline, and reduced quality of life affect patients’ independence and livelihood [7, 8]. Additionally, proximal femoral fractures are associated with a significant mortality risk during a hospital stay and following discharge. Hip “fracture fatality rate,“ or the proportion of patients who died following a hip fracture in a year, is an important health indicator in patients with this type of injury [9]. This index could be considered and monitored as the health system’s efficiency in dealing with elderly patients and geriatric care. The reported mortality rate of proximal femoral fractures in the literature ranges from 11% to more than 30% [10].

In line with the improvements in the prevention and medical care of proximal femoral fractures, orthopedic treatment has also undergone a dramatic shift in the past decades [11]. As our understanding of the biomechanics of these fractures has evolved, implants have been redesigned to ultimately improve the standards of care [12]. The appropriate implant selection is based on the access to the device, fracture characteristics, the patient’s bone quality and underlying medical conditions, and the surgeon’s preferences [12].

Although several large-scale studies have been performed in developed countries, there is a paucity in the literature regarding the outcomes of proximal femoral fractures in developing countries like Iran [13, 14]. Particularly, the incidence and risk factors of in-hospital and post-discharge mortality and functional outcomes are areas with minimal data available. Therefore, the goal of this study was to evaluate patients with a proximal femoral fracture at a level I tertiary referral trauma center in Iran, to determine the early and one-year mortality and its associated risk factors. Furthermore, we sought to evaluate functional outcomes of proximal femoral fractures at one year and the correlations with patients, injury, and treatment characteristics. The findings of this study could be implemented to improve the care and outcomes of elderly patients with this type of injury in future.

## Materials and methods

### Study design and population

In an IRB-approved retrospective cohort study, our institutional hospital information system (HIS) was queried for all patients admitted with a femoral neck or intertrochanteric fracture diagnosis from January 1, 2016, through December 31, 2018 to one referral orthopedic surgery center affialited with Kermanshah University of Medical Sciences, Kermanshah, Iran. Medical records were reviewed to include patients over 60 years of age who had a proximal femoral fracture and had a complete medical record and radiographs. The 60 years age cut-off for inclusion of the elderly population with proximal femoral fracture in this study was chosen based on the prevalence of this type of fracture in Iranian elederly population. Exclusion criteria included patients with pathological fractures, cancer under active treatment, follow-up loss, and patient access loss.

### Study variables and data collection

Basic patient information including sex, mechanism of injury, past medical history, American Society of Anesthesiologists (ASA) score status, the delay from fracture to surgery, type of anesthesia, surgical technique and the utilized hardware, the duration of hospital stay, and discharge status of patients were recorded. The admission radiographs were reviewed on the local picture archiving and communication system (PACS), and each fracture was classified according to the 2018 AO fracture and dislocation classification [15]. Associated fractures and follow-up radiographs were also extracted and reviewed. Anesthesia records were pre-operatively reviewed to extract the American Society of Anesthesiologists (ASA) score assigned to each patient by the attending anesthesiologist [16].

In-hospital and post-discharge mortalities at one and 12 months due to included types of fractures were identified and reviewed as the primary outcome in this study. Additionally, follow-up visits were reviewed to determine whether the patient recovered or died. The two time points of one-month and one-year for patient follow-up were chosen based on a literature review and similar studies in the field as most of the adverse outcomes following surgery of this condition in elderly population happen in the first month after surgery and also the first-year assessment is a crucial time-point in surgical outcomes investigations. Modified Harris Hip Scores (mHHS), collected at the latest clinic visit, were also recorded [17]. Furthermore, patients who did not have a post-operative visit at or after January 1, 2020, were called by phone between November 2020 and January 2021 to ascertain their health status. The mHHS form was completed over the phone by a trained investigator for these patients. The assessors who extracted data from charts and called patients were not involved in patient care and surgery. The treating surgeons were also not involved in data analysis.

### Statistical analysis

Statistical calculations and analyses were performed using SPSS Statistics for Windows, Version 25.0 (IBM). Statistically significant p for type I error was set at < 0.05. Descriptive statistics were used to calculate mean and standard deviation (± SD). Categorical variables were reported as frequency and percentage. Student’s t or Mann-Whitney U tests were used to compare means depending on the normality of data distribution. Pearson’s chi-square or Fischer’s exact test compared categorical variables. Kaplan-Meier survival analysis was performed to estimate the probability of survival at a specific time after the fracture (1-month and one-year periods), and the log-rank test was used to compare survival between groups. The Cox proportional hazard models were used to identify the predictors of mortality. Independent variables in the recruited models included age, sex, AO type of fracture, time from admission to surgery, orthopedic hardware utilized, and ASA score. Crude and adjusted hazard ratios with 95% confidence intervals (CI) were calculated for each variable.

### Ethical considerations

The study protocol was reviewed and approved by the Research Ethics Committee of Kermanshah University of Medical Sciences (IR.KUMS.REC.1398.1175). This study was performed following the Code of Ethics of the World Medical Association (Declaration of Helsinki) for experiments involving humans, and the human subjects’ privacy rights were respected.

## Results

### General findings

**Fig. 1:**
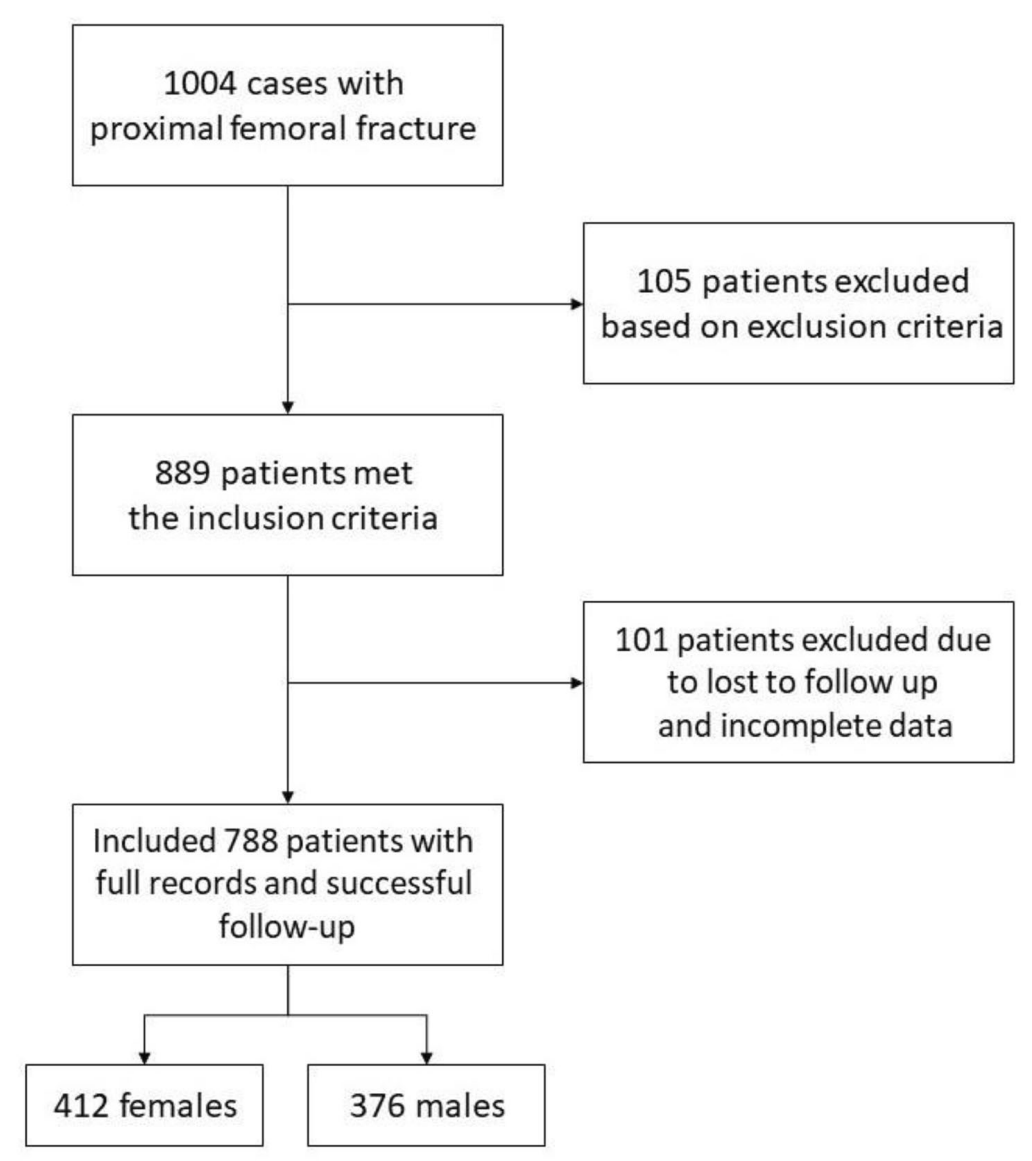
Flowchart of study population selection process

A total of 1004 cases with a proximal femoral fracture were identified during the study period. Of these, 889 patients satisfied the inclusion criteria. After excluding 101 (11.4%) patients who were lost to follow-up or had incomplete records/radiographs, a total of 788 patients including 412 females (52.3%) and 376 males (47.7%) with a female to male ratio of 1.1 were included in the final analysis (Fig. 1). The mean age of patients was 76.05 ± 10.01 (range, 60–111) years, and 341 patients (43.3%) were over 80 years old. No statistically significant difference was detected in the mean age between male and female patients (p = 0.24). Right-sided fractures were slightly more common, occurring in 409 patients (51.9%).

The majority of our patients (94%) sustained a fracture after a simple fall, and only 6% of fractures resulted from high-energy trauma. Concomitant fractures were diagnosed in 117 patients (15.0%), most commonly a distal radius or a humerus fracture. A history of previous hip fracture was present in 74 patients (9.5%). In 462 patients (59.4%), at least two comorbidities were present, the most common of which were dyslipidemia, diabetes mellitus, ischemic heart disease, and Alzheimer’s disease (Table 1).

**Table 1:** Basic demographics, injury, and outcome characteristics of included sample of populations in this study

Variable	N (%)
Number of patients	788
Age (years, mean ± SD)	76.05 ± 10.01(range: 60–111)
60–69	264 (33.5%)
70–79	183 (23.2%)
≥ 80	341 (43.3%)
Sex	
Male	376 (47.7%)
Female	412 (52.3%)
Laterality	
Left	379 (48.1%)
Right	409 (51.9%)
Comorbidities	
0–1	326 (41.4%)
≥ 2	462 (58.6%)
ASA Score	
1–2	565 (71.7%)
3–4	223 (28.3%)
Concomitant fractures	
Distal radius	62 (7.9%)
Humerus	34 (4.3%)
Other	21 (2.7%)
Total	117 (14.8%)
Previous hip fracture	74 (9.4%)
Time from fracture to surgery (days, mean ± SD)	3.80 ± 3.30 (range: 0–27)
Time from surgery to discharge (days, mean ± SD)	2.8 ± 2.68 (range: 2–11)
Mortality	
In-hospital	6 (0.8%)
One-month	45 (5.7%)
12-months	159 (20.2%)
Total	261 (33.1%)

### Fracture type

Among patients, 573 (72.7%) had an intertrochanteric fracture, while 215 (27.3%) had a femoral neck fracture. According to the AO classification, 180 fractures (22.8%) were considered type 31A1, 233 (29.6%) were 31A2, 160 (20.3%) were 31A3, and 215 (27.3%) were 31B. Patients with a femoral neck fracture were significantly younger than those with an intertrochanteric fracture (p = 0.01).

Overall, 23 patients (2.9%) did not undergo surgery and were treated non-operatively, with the remaining 765 patients (97.1%) received surgical treatment in one of the forms of screws, dynamic hip screws (DHS), open reduction and internal fixation (ORIF) with a proximal femoral plate, or bipolar hemiarthroplasty (Table 2).

**Table 2:** Treatment characteristics, broken down by fracture type

AO fracture type	31A1 (n = 180)	31A2 (n = 233)	31A3 (n = 160)	31B (n = 215)	Total (n = 788)	P-value
Age (years)	77.3 ± 9.5	76.9 ± 9.9	75.4 ± 10.4	74.4 ± 10.1	76.0 ± 10.0	**0.01**
Sex						0.58
Male	86 (10.9%)	111 (14.1%)	83 (10.5%)	96 (12.2%)	376 (47.7%)	
Female	94 (11.9%)	122 (15.5%)	77 (9.8%)	119 (15.1%)	412 (52.3%)	
Treatment						
DHS	75	83	22	-	180	
Bipolar hemiarthroplasty	44	40	28	207	319	
ORIF by plate	56	101	106	0	263	
Screw	0	0	0	3	3	
Non-operative	5	9	4	5	23	
Total	180 (22.8%)	233 (29.6%)	160 (20.3%)	215 (27.3%)	788	

*DHS: dynamic hip screw, ORIF: open reduction and internal fixation. Significant P-values are* ***bold***

### Mortality and survival

The mean follow-up period in this study was 33.31 months (range, 24–48), and survival assessment was determined based on health status on 1/1/2020. Overall mortality rate was 33.1% (262 patients), of which 6 deaths (2.3%) were in-hospital, 45 (17.2%) occurred during the first post-fracture month, and 159 (60.7%) happened during the first year following fracture. Although the mean overall survival was slightly higher in male patients (34.3 vs. 32.9 months, p = 0.009), men’s survival was lower in the ≥ 80-years age group (30.8 vs. 33.5 months).

Analysis of 1-month mortality showed a significant mortality difference in patients operated after 48 h of fracture (p = 0.01) and in patients with ASA scores of 3–4 compared to ASA scores of 1–2 (p = 0.001). One-year mortality data showed that the mortality rate in femoral neck fractures was lower compared to other types of fracture. Surgical delay of > 48 h, ASA scores of 3–4, and treatment by proximal femoral plate were associated with shorter survival (Table 3).

**Table 3:** Kaplan-Meier survival analysis for 1-month and 1-year mortality

	1-month mortality (n = 45)		1-year mortality (n = 159)	
	Number of deaths	P-value	Number of deaths	P-value
Gender		0.76		0.11
Male	21		67	
Female	24		92	
Age		0.15		0.12
60–69	9		44	
70–79	12		36	
≥ 80	24		79	
AO fracture type		0.52		**0.01**
31A1	12		43	
31A2	6		56	
31A3	9		32	
31B	8		28	
ASA Score		0.40		**0.02**
1–2	34		108	
3–4	10		51	
Delay to surgery		**0.01**		**0.002**
< 48 h	13		66	
2–7 days	31		88	
> 7 days	1		5	
Treatment		0.68		**0.002**
DHS	14		34	
Bipolar	17		45	
ORIF by plate	13		71	
Screw	0		1	
Non-operative	1		8	

*P values of the log-rank test are reported, and significant values are****bold***. *DHS: Dynamic hip screw, ORIF: Open reduction and internal fixation*

**Fig. 2:**
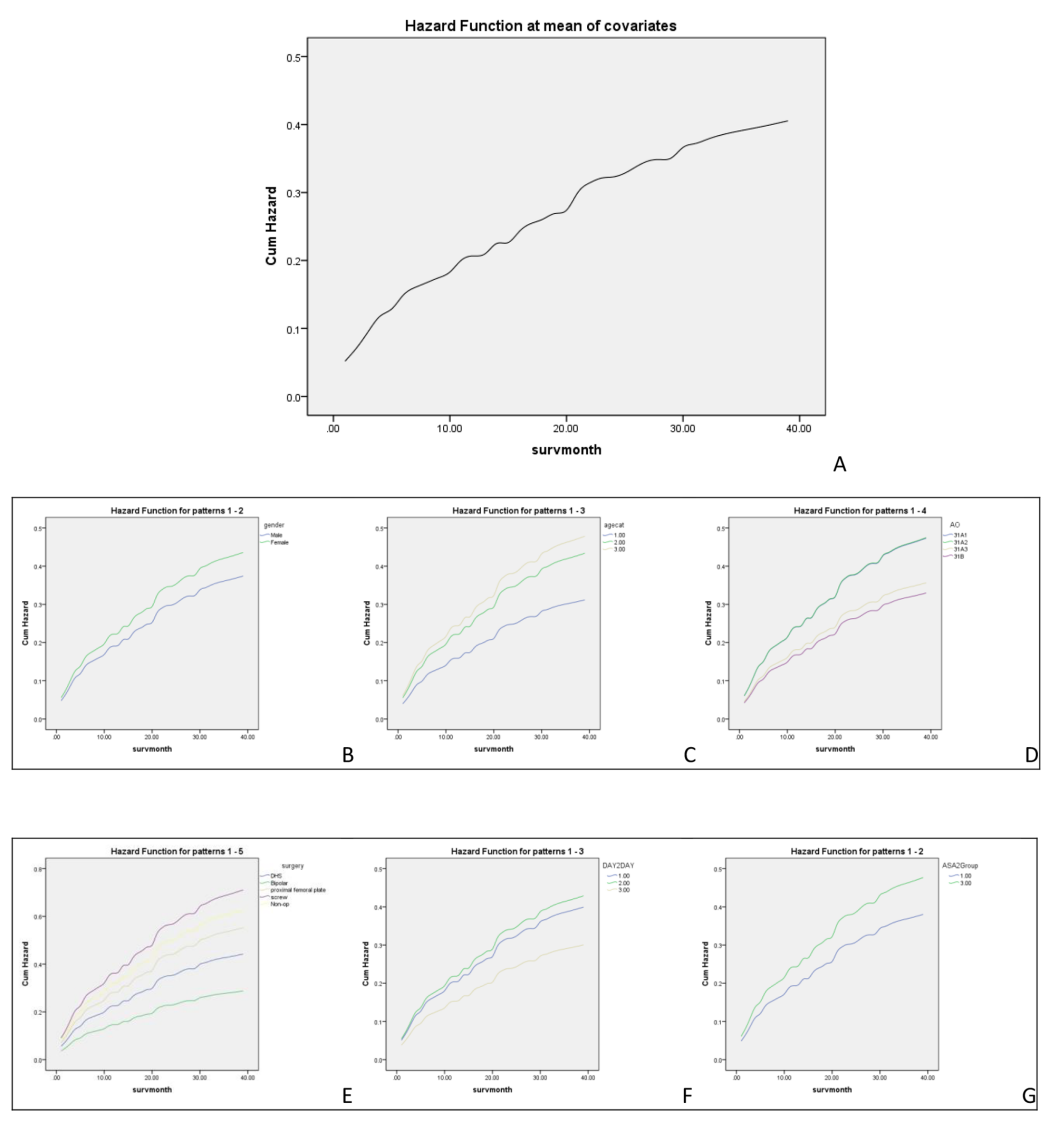
Cox regression Cumulative Hazard Ratio (CHR) curves in patients with proximal femoral fracture for each variable. A: CHR for all variable, B: CHR for Sex, C: CHR for each age category (1=60-70 Years, 2= 70-80, 3= >80), D: CHR for each AO fracture type, E: CHR based on surgical device, F: CHR based on time of admission to surgery (1= <48-hour, 2= 2–7-day, 3= >1 week), G: CHR for ASA score (1 = score 1 and 2, 3 = score 3,4)

Univariate Cox regression survival analysis revealed that femoral neck fractures had a significantly lower mortality risk compared to the intertrochanteric fractures (p = 0.001). Additionally, delayed surgery (p = 0.02) and an ASA score of 3–4 (p = 0.04) significantly increased the mortality risk, while treatment with bipolar hemiarthroplasty decreased the risk of mortality (p = 0.001). On multivariable Cox regression analysis, age ≥ 80 years, ORIF with a proximal femoral plate, delayed surgery, and ASA scores of 3–4 were risk factors of mortality, while bipolar hemiarthroplasty reduced the risk of mortality (Fig. 2). Table 4 summarizes the results of Cox regression analysis.

**Table 4:** Risk factors of mortality according to the univariate and multivariable-adjusted Cox regression analysis

	Univariate			Multivariable		
	Hazard Ratio	95% CI	P-value	Hazard Ratio	95% CI	P-value
Gender						
Female	1.13	0.88–1.44	0.31	1.16	0.90–1.49	0.22
Age						
70–79	1.76	0.73–4.25	0.20	1.39	0.98–1.96	0.06
≥ 80	2.06	0.96–4.44	0.64	1.53	1.13–2.06	**0.005**
AO type						
31A2	1.04	0.76–1.41	0.78	1.00	0.73–1.37	0.98
31A3	0.78	0.54–1.12	0.19	0.75	0.51–1.10	0.14
31B	0.45	0.31–0.66	**0.001**	0.69	0.42–1.13	0.14
Treatment						
Bipolar	0.53	0.38–0.74	**0.001**	0.65	0.42–0.99	**0.04**
ORIF by plate	1.14	0.84–1.54	0.39	1.25	1.11–1.80	**0.04**
Screw	0.92	0.12–6.56	0.92	1.60	0.20–12.4	0.65
Non-operative	1.51	0.81–2.79	0.18	1.41	0.72–2.77	0.31
Delay to surgery						
2–7 day	1.11	1.16–1.43	**0.02**	1.17	1.02–1.40	**0.03**
> 7 days	0.42	0.87–1.36	0.12	1.05	0.53–1.28	0.23
ASA score						
3–4	1.11	1.08–1.47	**0.04**	1.25	1.05–1.64	**0.04**

*The presented data is compared to the baseline category as reference for each comparison, which is not shown. Significant P-values are in* ***bold***

A separate multivariable Cox regression analysis was performed to evaluate the risk factors of mortality among intertrochanteric fractures only (AO type 31B cases were excluded). The results indicated that a bipolar hemiarthroplasty decreased the risk of mortality with a hazard ratio of 0.61 (95% CI: 0.39–0.95, p = 0.02), while age ≥ 80 years, delayed surgery, ORIF by a proximal femoral plate, and ASA scores of 3–4 were significant risk factors of mortality (Table 5).

**Table 5:** Risk factors of mortality in intertrochanteric fractures according to multivariable-adjusted Cox regression analysis

	Multivariable Cox Regression		
	Hazard Ratio	95% CI	P-value
Sex			
Female	1.07	0.82–1.40	0.59
Age			
70–79	1.35	0.92–1.96	0.11
≥ 80	1.40	1.01–1.94	**0.04**
AO type			
31A2	1.00	0.74–1.37	0.95
31A3	0.73	0.50–1.06	0.73
Treatment			
Bipolar	0.61	0.39–0.95	**0.02**
ORIF by plate	1.34	1.11–1.70	**0.01**
Screw	1.60	0.80–3.21	0.80
Delay to surgery			
2–7 day	1.02	1.00–1.35	**0.01**
> 7 days	1.05	0.60–1.41	0.34
ASA score			
3–4	1.11	1.05–1.27	**0.04**

*The presented data is compared to the baseline category as reference for each comparison, which is not shown. Significant P values are in* ***bold***

### Functional outcomes

The results of the mHHS scores at follow-up are listed in Table 6. The overall mean mHHS score was 53.80 ± 20.78. Patients with a femoral neck fracture (56.14 ± 21.34, p = 0.005), treatment with bipolar hemiarthroplasty (56.46 ± 21.01, p = 0.001), and DHS (54.68 ± 19.80, p = 0.001) had significantly higher mHHS scores, while patients with a type 31A3 fracture (50.06 ± 1.57, p = 0.005) and those who underwent ORIF with a proximal femoral plate (50.91 ± 19.92, p = 0.001) had a significantly lower mean mHHS. No statistically significant difference was detected among age groups (p = 0.651) and between two sexes (p = 0.412).

**Table 6:** Functional outcomes, according to HHS, are broken down by subscales and treatment

AO fracture type		Pain	Stair	Limp	Show	Walking	Transport	Walk aid	Total
31A1	Bipolar	35.8	1.8	6.5	2.7	4.9	1.5	6.1	
	DHS	32.0	1.5	6.5	3.0	4.5	1.5	6.0	
	ORIF	30	1.2	5.1	2.9	4.1	1.7	4.5	
	Non-op	30	1.4	4.6	3.2	2.2	2	4.4	
	Overall	32.2	1.5	6.0	2.9	4.4	1.6	5.5	**54.4**
31A2	Bipolar	33.1	1.4	6.6	2.8	4.2	1.5	4.7	
	DHS	33.5	1.5	6.3	2.7	4.3	1.5	5.4	
	ORIF	30.9	1.4	5.3	3.0	4.9	1.6	5.1	
	Non-op	26.6	1.7	4.4	3.7	3.6	1.7	5.5	
	Overall	32.0	1.4	5.8	2.9	4.5	1.5	5.2	**53.7**
31A3	Bipolar	33.0	1.2	4.8	2.7	4.5	1.7	5.6	
	DHS	29.8	1.7	5.0	2.4	3.6	1.4	5.2	
	ORIF	29.6	1.5	5.7	2.8	3.9	1.6	4.7	
	Non-op	17.5	0.5	1.2	2	1	2	2.5	
	Overall	29.9	1.4	5.3	2.7	3.9	1.6	4.9	**50.0**
31B	Bipolar	34.5	1.5	5.6	2.7	4.8	1.5	5.4	
	Non-op	32	1.8	5.8	3.2	3.8	1.8	3	
	Screw	21.3	2	4.3	2	2.3	1.6	5.3	
	Overall	34.3	1.5	5.6	2.7	4.8	1.5	5.4	**56.1**
**Total**		**32.3**	**1.5**	**5.7**	**2.8**	**4.4**	**1.6**	**5.2**	**53.8**

## Discussion

As the global life expectancy rises and the populations are aging, the incidence of fragility fractures, particularly hip fractures, is increasing worldwide. While preventive measures are being implemented, the evidence clearly shows that hip fractures are a global health challenge. This study was performed to establish the mortality rates for hip fractures in the elderly in Iran, besides the patient-, injury-, and treatment-related risk factors of early and mid-term mortality. Finally, we aimed to evaluate the functional outcomes of hip fractures in elderly population residing in Iran. The main findings of this study were the significant contribution of patients’ age, type of fracture, time of receiving surgical treatment, and type of surgical repair to the mortality following proximal femoral fractures.

We collected data from 788 patients aged over 60 years old who were treated for a hip fracture at a referral trauma center in Iran. Although several studies have been performed in the developing world on hip fracture mortality, the common limitation of the majority is the high loss to follow-up rate. This common shortage might be largely the result of the limited access to healthcare and insurance [18, 19]. We attempted to mitigate this limitation by calling the patients with less than a year of follow-up to ascertain the patient’s health status and determine their functional outcomes according to the mHHS. After this study, we have had a largely positive experience with virtual clinic visits implemented in response to the COVID-19 pandemic and have continued the practice [20, 21]. With this approach, only about 11% of the total patient population was unavailable at one year, increasing our results’ validity.

It is well-established that women have a higher risk of hip fractures, with a female to male ratio of 1.7 to 2.5 in the literature [22, 23]. Interestingly, studies from the Middle East region have reported a much closer incidence between males and females, ranging from 0.9 to 1.4 [24, 25], which has been replicated in previous studies from Iran, at 1.1 [26, 27]. We also found a female to male ratio of 1.1 in our patients. Although this was not an epidemiologic study, our findings in line with the findings of previous studies, call the need for epidemiologic studies to determine the underlying determinants of these results.

The 1-month mortality has been reported about 3–14% in the literature. The large mortality range is partly explained by the baseline patient and injury characteristics. Regardless, we found a 1-month mortality rate of 5.7%, which is in line with the literature. Of note, only six in-hospital mortalities were recorded. We also found a 1-year mortality rate of 20.2%.

Interestingly, older studies have reported higher mortality rates of about 21–39% [28, 29], while more recent studies have reported a mortality rate of about 2.5–14.6% [30, 31], which suggests a trend of decreasing mortality with improved care. Additionally, at a mean follow-up of 33 months, we found a 33.1% mortality rate. Although this is not a standard time point for reporting mortality, it is imperative to appreciate that a third of patients with a fragility hip fracture die during the first three years after fracture and implement measures to decrease this alarmingly high rate. It should be noted that if we had not called the patients to assess their health status, many of these patients would have been assumed alive or lost to follow-up. Therefore, studies on long-term mortality of hip fractures should strive to minimize their loss to follow-up rate.

We performed a Kaplan-Meier survival analysis with log-rank tests to compare survival between groups, which showed that a > 48-hour delay to surgery was associated with a significantly higher 1-month mortality. The principal factors contributing to surgical delays in these patients include the unavailability of surgical personnel and instruments, the necessity for thorough preoperative investigation and evaluation of elderly patients, as well as the requirement for medical stabilization [32]. Risk factors of 1-year mortality were > 48-hour delay to surgery, AO fracture type, ASA scores of 3–4, and treatment with a proximal femoral plate. Cox regression analysis was also performed to characterize the risk factors of mortality during the follow-up period. On univariate Cox regression, femoral neck fractures and treatment with bipolar hemiarthroplasty significantly decreased the risk of 1-year mortality, while delayed surgery and an ASA scores of 3–4 significantly increased the risk. On a multivariable Cox regression model; however, age ≥ 80 years, the use of a proximal femoral plate, delayed surgery, and an ASA score of 3–4 were significant risk factors of mortality at one year, while a bipolar hemiarthroplasty was a protective factor. Previous studies have also reported that femoral neck fractures have a lower mortality rate than intertrochanteric fractures and also that plates have a worse outcome than intramedullary devices [33].

While joint replacement is a standard treatment of femoral neck fractures, its use is not common in intertrochanteric fractures. In order to minimize the effect of the femoral neck fractures in the Cox regression model and specifically evaluate the risk factors of mortality in intertrochanteric fractures, we performed a separate Cox regression analysis on this subset of patients. We found that age ≥ 80 years, a delayed surgery, ASA scores of 3–4, and the use of a proximal femoral plate were risk factors of mortality, while bipolar hemiarthroplasty was a protective factor in intertrochanteric fractures. The AO fracture type and age were not risk factors of mortality. Several studies have reported favorable arthroplasty outcomes in intertrochanteric fractures with decreased mortality [34, 35]. However, there are studies linking hemiarthroplasty with increased mortality [36]. Although controversy exists, hemiarthroplasty seems to provide earlier weight-bearing and a lower reoperation rate.

The common risk factor of mortality in all of our survival analyses was using a proximal femoral plate. Although plates showed promising results in earlier reports [37], several studies show poor biomechanical properties of proximal femoral plates [38]. Additionally, a longer operation time with a higher complication rate and a delayed return to walking are other drawbacks of the device [39]. Therefore, with the results of this study, we have abandoned the use of proximal femoral plates in the treatment of intertrochanteric fractures, although we still utilize them in young patients with certain peritrochanteric fractures.

We also assessed the functional outcomes of hip fractures with the mHHS. Interestingly, patients with a femoral neck fracture, treatment with a bipolar hemiarthroplasty, and the use of a DHS were associated with a significantly higher mHHS. In contrast, AO type 31A3 and the use of a proximal femoral plate were associated with lower scores.

We acknowledge several limitations to our study. First, this is a retrospective study, and therefore, we had no control over treatments. Second, several surgeons were responsible for our patients, and therefore, the treatment decisions were not standardized and heterogeity in surgical outcomes was inevitable. In addition, being situated in a developing country like Iran, the surgical approach and orthopedic hardware are selected based on the patient and injury characteristics and according to the availability of each hardware and surgeon’s preferences. This is important because while intramedullary nails are preferred over bipolar hemiarthroplasty and proximal femoral plates in the literature, we did not have access to these devices during the study period. Another limitation of the current study was tha lack of data on comorbidities of the included patients which limited the analysis based on associated medical conditions that we know are highly prevalent among elderly population.

Furthermore, our focus in this study was to report the mortality rate and functional outcomes, especially compared between different surgical procedures. Therefore, we did not report other complications (e.g., infection), which are also important in making treatment decisions and the patients’ long-term function. Despite these limitations, we have studied a large cohort of patients with hip fractures, and with minimal loss to follow-up, managed to report mortality up to 1 year after fracture with the underlying risk factors. Reporting the functional outcomes in addition to the mortality rate is another strength of this study.

## Conclusion

To conclude, in the study of 788 Iranian elderly patients with a hip fracture, we found 1-month and 1-year mortality rates 5.7% and 20.2%, respectively. We found several risk factors of mortality, including age ≥ 80 years, a > 48-hour delay to surgery, and pre-operative ASA scores of 3–4. Furthermore, the use of a proximal femoral plate was a significant risk factor for mortality and lower mHHS scores, while a bipolar hemiarthroplasty was associated with a decreased risk of mortality and higher mHHS scores.

## Data Availability

The datasets analyzed in the current study are available from the corresponding author upon reasonable request.

## Abbreviations

CHR: Cox regression Cumulative Hazard Ratio
mHHS: Modified Harris Hip Scores
ASA: American Society of Anesthesiologists
HIS: hospital information system
PACS: picture archiving, and communication system
±SD: standard deviations
CI: confidence intervals
DHS: dynamic hip screws
ORIF: open reduction and internal fixation
